# Comparison of Decompression Alone Versus Decompression with Fusion for Stenotic Lumbar Spine: A Systematic Review and Meta-analysis

**DOI:** 10.7759/cureus.3135

**Published:** 2018-08-13

**Authors:** Syed Ijlal Ahmed, Gohar Javed, Syeda Beenish Bareeqa, Ali Shah, Maha Zubair, Rabbia Faisal Avedia, Noor Rahman, Syeda Sana Samar, Kashif Aziz

**Affiliations:** 1 Graduate Student, Liaquat National Hospital and Medical College, Karachi, PAK; 2 Neurosurgery, Aga Khan University and Hospital, Karachi, PAK; 3 Medical Student, Jinnah Medical and Dental College, Karachi, PAK; 4 Medical Graduate, Dow University of Health Sciences, Karachi, PAK; 5 Miscellaneous, Ziauddin Medical College, Karachi, PAK; 6 Miscellaneous, Ziauddin Medical University, Karachi, PAK; 7 Medical Student, Jinnah Sindh Medical University, Karachi , PAK; 8 Internal Medicine, Icahn School of Medicine at Mount Sinai Queens Hospital Center, New York, USA

**Keywords:** spinal stenosis, decompression surgery, fusion surgery, oswestry disability index

## Abstract

The first line of treatment for lumbar spinal stenosis (with or without lumbar degenerative spondylolisthesis) involves conservative options such as anti-inflammatory drugs and analgesics. Approximately, 10%-15% of patients require surgery. Surgical treatment aims to decompress the spinal canal and dural sac from degenerative bony and ligamentous overgrowth. Different studies have given conflicting results. The aim of our study is to clear the confusion by comparing two surgical techniques. This meta-analysis was conducted in accordance with the preferred reporting items for systematic reviews and meta-analysis (PRISMA) guidelines. A literature search was conducted of the Ovid Embase, Scopus, Pubmed, Ovid Medline, Google Scholar, and Cochrane library databases. A quality and risk of bias assessment was also done. The analysis was done using Revman software (The Nordic Cochrane Centre, The Cochrane Collaboration, 2014, Copenhagen, Denmark).

A total of 76 studies were extracted from the literature search and 29 studies with relevant information were shortlisted. Nine studies were included in the meta-analysis after a quality assessment and eligibility. Fusion with decompression surgery was found to be a better technique when compared to decompression alone for spinal stenosis in terms of the Oswestry Disability index and the visual analog pain scale for back and leg pain. On the basis of the meta-analysis of the recent medical literature, the authors concluded that decompression with fusion is a 3.5-times better surgical technique than decompression alone for spinal stenosis.

## Introduction and background

Lumbar spinal stenosis (LSS) is defined as the narrowing of the lumbar spinal canal and nerve root canals due to hypertrophic lesions of the facet joints, ligamentum flavum, and inter-vertebral discs, which leads to a debilitating compression of the spinal nerves and blood vessels [1-2]. It is one of the most prevalent spinal disorders in the elderly. Its incidence appears to be rising and it is estimated that by the year 2025, about 64-million elderly will be affected by it [3]. LSS often occurs in combination with lumbar degenerative spondylolisthesis (LDS), which, in turn, is defined as the slipping forward of one lumbar vertebra on another with an intact neural arch [4]. The first line of treatment for LSS (with or without LDS) involves conservative options, such as anti-inflammatory drugs and analgesics. Approximately 10%-15% of patients develop an incapacitating back and/or leg pain (BP and/or LP), which requires surgery [5]. Surgical treatment aims to decompress the spinal canal and dural sac from degenerative bony and ligamentous overgrowth [6]. Conventional surgical options include decompression (D) and decompression plus fusion (D+F) [7]. The aim of lumbar decompression is to decompress the neural elements while preserving stability [8]. If the lumbar spine is unstable, an instrumented fusion is performed in addition. The immobilization of one or more motion segments by fusion techniques involves joining two or more vertebrae permanently into one solid bone with no space between them [9].

Different studies have given conflicting results with some finding that decompression alone was associated with more favorable outcomes in spinal stenosis measure (SSM) scores and others demonstrating better outcomes with the added effect of fusion [6,10]. Evidence indicates that patients with lumbar spinal stenosis, without deformity or instability, treated with wide decompression, may suffer from iatrogenic lumbar instability [7].

A recent meta-analysis published by Chen et al compared D and D+F in patients with LDS. The study concluded that D+F did not yield better clinical outcomes than D alone [11]. Our study aims to assess the additive effect of fusion on decompression surgery and to ascertain if D+F is more effective than D alone in patients with LSS (with/without LDS).

## Review

Materials and methods

Literature Search Strategy

We conducted this meta-analysis in accordance with the preferred reporting items for systematic reviews and meta-analysis (PRISMA) guidelines. A detailed literature search was conducted by two independent authors using the keywords “spinal fusion surgery,” “spinal decompression surgery,” “spinal stenosis,” “Oswestry Disability index,” and “degenerative spondylolisthesis” to search the Ovid Embase, Scopus, Pubmed, Ovid Medline, Google Scholar and Cochrane library databases. Relevant terms or synonyms other than keywords were utilized to conduct a comprehensive search in accordance with the pre-specified eligibility criteria. All the searched articles were exported and cited through Endnote. The search strategy was limited to medical literature in English, published from 2012 to 2018. In cases of an unavailability of the full text or incomplete data, the corresponding author was contacted.

Eligibility Criteria

The study types included in our research were randomized controlled trials and both prospective and retrospective cohort studies. However, case reports, letters to the editor, commentaries, cross-sectional surveys, and documentaries were excluded but used only to bridge and link the outcomes of our study with past medical research for discussion. Moreover, studies in non-English literature, studies which assessed the outcome in pathologies other than intracranial aneurysms, studies without definitive numbers or values, experimental animal trials, and studies with figurative or graphical results presentation without any particular numerical values were also excluded from this research. Two independent authors retrieved the required data in accordance with the mentioned eligibility criteria. Any disagreement was resolved by a collaborative discussion.

Data Collection

Studies were assessed and data were extracted by two independent reviewers according to PRISMA guidelines. Data were collected and compiled on a predefined evidence table. Articles were selected on the basis of relevance to the topic, appropriate sample size, sampling technique, and randomization. The collected data include author, year of publication, sample size, study design, Oswestry disability index (ODI), visual analog pain score (VAS) for back pain, VAS for leg pain, and the statistical results of the study (relative risk, confidence interval, and p-value). Any disagreement was resolved with a collaborative consensus.

Quality Assessment and Risk of Bias

To assess the quality of extracted data, the Newcastle-Ottawa scale was used for cohort studies and the Jadad scale (also known as the Oxford quality scoring system) was utilized for randomized controlled trials. To avoid any risk of bias, we evaluated data using the Cochrane risk of bias tool, which analyzed the data in terms of allocation concealment, blinding, sequence generation, selective outcome reporting, and incomplete outcome data.

Data Analysis and Primary Outcomes

The data was entered on RevMan version five (The Nordic Cochrane Centre, The Cochrane Collaboration, 2014, Copenhagen, Denmark) and analyzed using a forest plot for a visual estimation of the meta-analysis. The test for heterogeneity was also done (p-value <0.05). A fixed effect model with the inverse variance method was used to obtain the overall mean difference estimates and the 95% confidence interval (CI) in order to assess the effect of fusion surgery with decompression and decompression surgery alone on the Oswestry disability index and visual analog scale for back and leg pain. A p-value of less than 0.05 was considered significant. 

Results

Extracted Studies Characteristics

We selected 76 articles (which included cohorts, case-control studies, randomized trials, and reviews) on the basis of relevant titles and abstracts after a systematic review. After going through the abstracts of selected articles, 19 duplicates were excluded from the selected pool. The remaining 57 articles were screened for the required information. Another pool of 28 articles was removed after a screening of titles and abstracts. Removal was on the basis of non-English language literature and the unavailability of the full text of articles. The balance 29 full-text articles were assessed in accordance with the eligibility criteria out of which nine articles were finalized for quantitative synthesis [12-20].

Overall Outcomes

**Figure 1:**
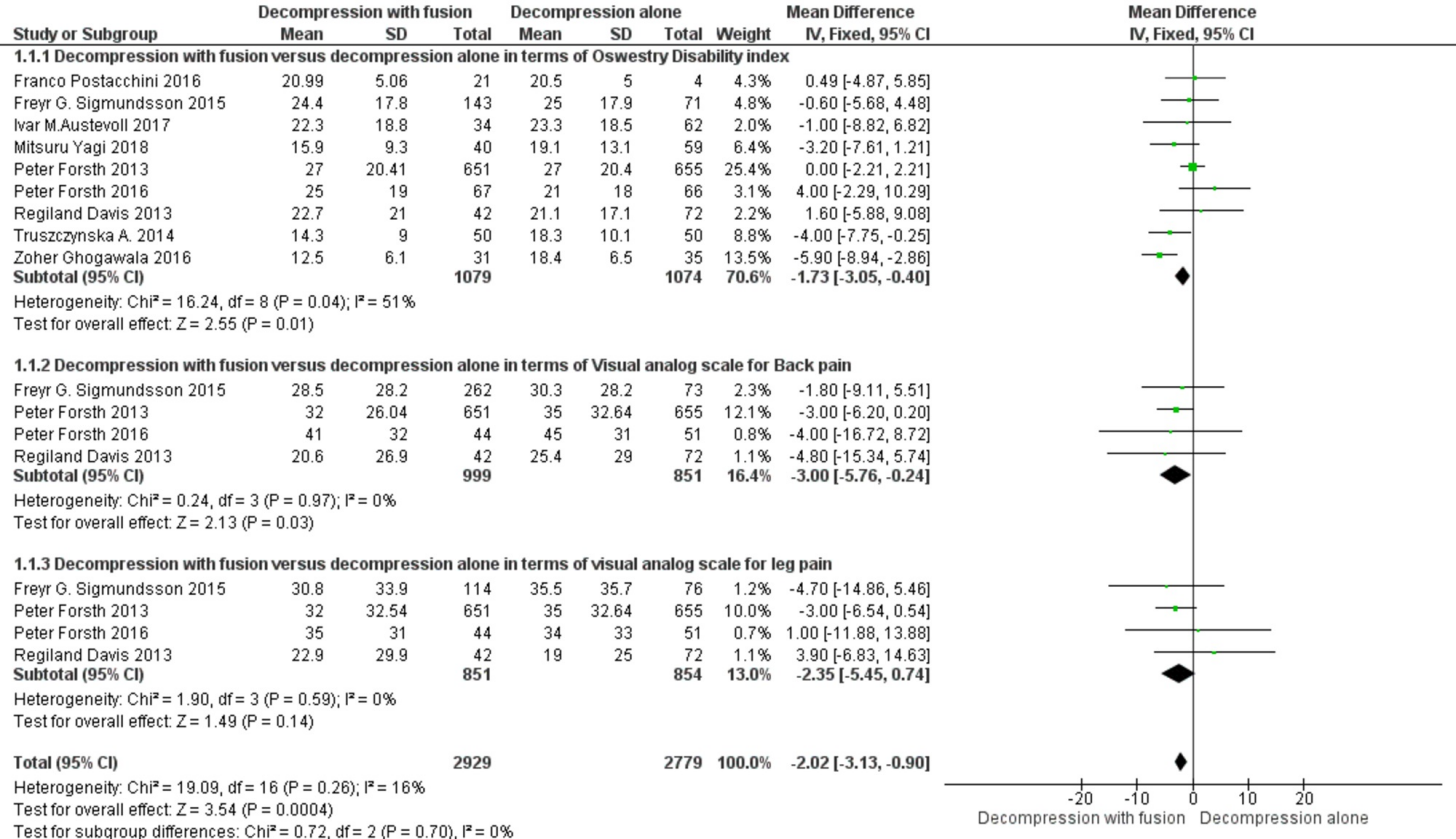
Descriptive representation of the meta-analysis through a forest plot

**Figure 2:**
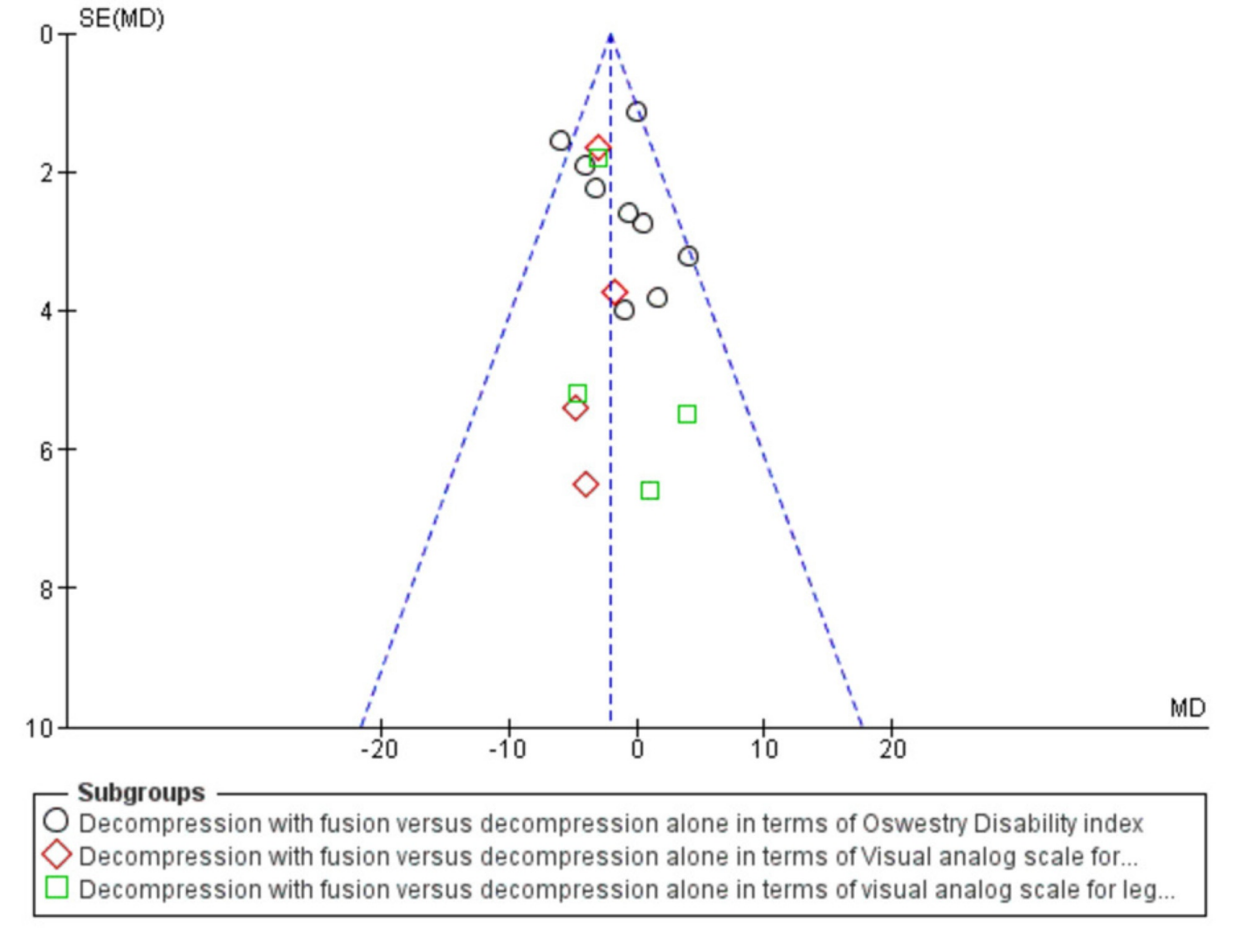
Presentation of heterogenicity through a funnel plot

A total of 2929 patients were analyzed in the fusion with decompression group while 2779 patients were analyzed in the decompression group. Decompression with fusion was found to be 2.55 times better as compared to decompression alone in terms of the Oswestry Disability index (mean difference -1.73, CI 95%, {-3.05, -0.40}). The decompression with fusion group was also found 2.1 times (p=0.01) superior than the decompression alone group in terms of visual analog pain scale for back pain (mean difference -3.0, CI 95%, (-5.76, -0.24)) and 1.4 times (p=0.03) superior for leg pain (mean difference -2.35, CI 95%, (-2.35, 0.740)). The overall effect showed fusion with decompression as a 3.54 times better surgical technique over decompression alone for spinal stenosis (p=0.0004) (mean difference -2.02, CI 95%, (-3.13, -0.90)). The detailed meta-analysis forest plot is shown in Figure 1. The heterogeneity test was also performed and the overall heterogeneity in the meta-analysis was only 16% (Chi-square=19.09, difference=16, p-value=0.14). The funnel plot for heterogeneity is shown in Figure 2.

Discussion

Our meta-analysis compared the outcome of D versus D+F in terms of ODI, VAS (leg pain and back pain). Studies conducted in the past suggest that a radiographically detectable instability of the posterior elements of the spine require fusion surgery along with decompression for favorable postoperative outcomes [21-22]. Similarly, in our meta-analysis, we found fusion with decompression to be better than decompression alone in terms of ODI index and visual analog pain scale.

However, in contrast to our findings, a meta-analysis study showed that the addition of fusion did not result in improved clinical outcomes compared to D over a follow-up period of two years [23]. Similar to our findings, Li et al. concluded that D+F showed less favorable outcomes in terms of ODI, length of hospital stay, and blood loss in comparison with D alone (using coflex). They did not find any significant difference in VAS and major device-related complications, which is in contrast to our findings [24]. Another international study showed that both the D and D+F groups resulted in reduced VAS LP and BP [25]. Aihara et al. reported higher postoperative scores in the D alone group. However, their results were not statistically significant. Also, less blood loss and shorter post-operative hospitalization were observed in the D alone group [26].

Similar to our findings, high rates of satisfaction and decreased leg pain scores were observed in patients with lumbar degenerative spondylolisthesis, who underwent D+F rather than D alone [27]. However, another study suggested that the degree of satisfaction two years after surgery was slightly higher in patients who underwent D alone [28].

## Conclusions

On the basis of a meta-analysis of the recent medical literature, authors concluded that decompression with fusion is a 3.5 times better surgical technique compared to decompression alone for spinal stenosis in terms of Oswestry disability index and visual analog pain scale for back pain and leg pain.
